# Analysis of Post-Deployment Cognitive Performance and Symptom Recovery in U.S. Marines

**DOI:** 10.1371/journal.pone.0079595

**Published:** 2013-11-27

**Authors:** F. J. Haran, Aimee L. Alphonso, Alia Creason, Justin S. Campbell, Dagny Johnson, Emily Young, Jack W. Tsao

**Affiliations:** 1 Biomedical Research & Operations Department, Navy Experimental Diving Unit, Panama City Beach, Florida, United States of America; 2 Henry M. Jackson Foundation for the Advancement of Military Medicine, Arlington, Virginia, United States of America; 3 Department of Orthopedics and Rehabilitation, Walter Reed National Military Medical Center, Bethesda, Maryland, United States of America; 4 Universal Consulting Services, Inc., Arlington, Virginia, United States of America; 5 Traumatic Brain Injury Programs, Wounded, Ill, and Injured Directorate, U.S. Navy Bureau of Medicine and Surgery (BUMED), Arlington, Virginia, United States of America; 6 Naval Space and Warfare Systems Center Pacific, San Diego, California, United States of America; 7 Eyak Corporation, Arlington, Virginia, United States of America; Max Planck Institute of Psychiatry, Germany

## Abstract

**Background:**

Computerized neurocognitive testing (NCAT) has been proposed to be useful as a screening tool for post-deployment cognitive deficits in the setting of mild traumatic brain injury (mTBI). We assessed the clinical utility of post-injury/post-deployment Automated Neurocognitive Assessment Metric (ANAM) testing, using a longitudinal design to compare baseline ANAM tests with two post-deployment ANAM tests in a group of Marines who experienced combat during deployment.

**Methods and Findings:**

Post-deployment cognitive performance and symptom recovery were compared in a subsample of 1324 U.S. Marines with high rates of combat exposure during deployment. Of the sample, 169 Marines had available baseline and twice repeated post-deployment ANAM results. A retrospective analysis of the ANAM data, which consisted of a self-report questionnaire about deployment-related blast exposure, recent history of mTBI, current clinical symptoms, and cognitive performance. Self-reported concussion sustained anytime during deployment was associated with a decrease in cognitive performance measured between 2–8 weeks post-deployment. At the second post-deployment test conducted on average eight months later, performance on the second simple reaction time test, in particular, remained impaired and was the most consistent and sensitive indicator of the cognitive decrements. Additionally, post-concussive symptoms were shown to persist in injured Marines with a self-reported history of concussion for an additional five months after most cognitive deficits resolved. Results of this study showed a measurable deployment effect on cognitive performance, although this effect appears to resolve without lasting clinical sequelae in those without history of deployment-related concussion.

**Conclusions:**

These results highlight the need for a detailed clinical examination for service members with history of concussion and persistent clinical symptoms. Reliance solely upon computerized neurocognitive testing as a method for identifying service members requiring clinical follow-up post-concussion is not recommended, as cognitive functioning only slowly returned to baseline levels in the setting of persistent clinical symptoms.

## Introduction

Since October 2001, over two million service members (SMs) have deployed in support of Operation Iraqi Freedom (OIF) and Operation Enduring Freedom (OEF) in support of the North Atlantic Treaty Alliance (NATO) International Security Assistance Force (ISAF) in Afghanistan[1]. One of the greatest risks that service members face during deployment is exposure to injury as a result of explosive blasts (e.g., mortar shells, rocket-propelled grenades, landmines, and improvised explosive devices [IEDs]), which have caused approximately 78% of all combat-related injuries in OIF/OEF and 40% of military deaths in OIF[2], [3]. Traumatic Brain Injury (TBI) has become a common combat-related injury secondary to blast exposure. The United States (U.S.) Department of Defense (DoD) defines traumatic brain injury (TBI) as a “traumatically induced structural injury and/or physiological disruption of brain function as a result of an external force”[4].

Mild TBI (mTBI), commonly referred to as concussion, is the most prevalent form of TBI and is recognized as an alteration of consciousness ≤24 hours, loss of consciousness (if any) <30 minutes, posttraumatic amnesia ≤24 hours, and normal structural neuroimaging[4], [5]. The symptoms of mTBI can be somatic (e.g., headache, dizziness, sensitivity to light and sound); cognitive (e.g., difficulty with attention, memory, and language); and/or psychological (e.g., irritability, depression, anxiety)[4]. Recent estimates suggest that 7.4% to 22.8% of SMs return from deployment having sustained TBI of any severity [6], [7]. Given the prevalence of mTBI in the warfighter, the military healthcare system has mandated military Services to have screening protocols in place to assist with determining if follow-up clinical evaluation by a medical specialist. All SMs are required to take a pre-deployment neurocognitive assessment as a baseline; however, the DoD does not have a policy requiring post-deployment testing for all SMs. SMs who answer affirmatively on post-deployment TBI screening questions which are part of the DoD required Post-Deployment Health Assessment (PDHA), used to identify SMs who need further clinical evaluation as a result of sustaining a mTBI/concussion during deployment, may receive post-deployment neurocognitive testing as part of this clinical evaluation.

The designated DoD tool for neurocognitive assessment is the Automated Neuropsychological Assessment Metrics version 4 TBI Military battery (ANAM4 TBI-MIL). The ANAM4 TBI-MIL is a self-administered, computerized test that measures neurocognitive performance in six domains: reaction time, processing speed, learning, working memory, delayed memory, and spatial memory [8], [9]. The ANAM4 TBI-MIL has been shown to be valid and have clinical utility as an individual diagnostic or population screening tool for the detection of neurocognitive dysfunction following a single, uncomplicated concussion within an acute post-injury (i.e., 72 hours) window, particularly if the results are compared against a pre-deployment/pre-injury baseline [10], [11], [12], [13].

Recent research has shown that the ANAM4 TBI-MIL may have clinical utility as an indicator of neurocognitive recovery when given outside of the acute window. Reaction time based performance tests have been reported to be correlated with to return-to-duty time with poorer performance linked with longer time needed to return to duty [14]. Bryan and Hernandez reported that SMs with a history of mTBI had a greater decrease in neurocognitive performance when compared to a pre-injury/pre-deployment baseline than SMs without a history of mTBI [11]. The study population was tested up to two years after injury indicating that some SMs may have persistent long-term cognitive deficits following TBI and that the ANAM4 TBI-MIL is sensitive even years post-injury [11].

The aim of this study is to assess the clinical utility of post-injury/post-deployment ANAM testing, including as a possible measure of neurocognitive change, using a longitudinal design to compare baseline ANAMs with two post-deployment ANAMs taken from a sample of Marines who experienced combat during their deployment.

## Materials and Methods

### Ethics

This project was part of a Navy Medicine performance improvement effort examining the utility of enhanced post-deployment TBI screening, including post-deployment ANAM testing. This study was approved by the Naval Air Warfare Center Aircraft Division (NAWCAD) Institutional Review Board (IRB), Patuxent River, MD (Protocol #: NAWCAD.2011.0003-CR01-EMC). The study was a retrospective analysis of de-identified data and did not involve any interaction with human subjects, thus, informed consent was waived by the NAWCAD IRB.

### Subjects

A convenience sample of de-identified data (N = 1324) were obtained from Marine Corps units with known high rates of concussion and/or combat and blast exposure while on deployment who returned to their home bases between February 2010 and December of 2011.

Inclusion criteria required that all SMs completed three ANAM4 TBI-MIL assessments (i.e., a pre-deployment, an initial or 1^st^ post-deployment, and a 2^nd^ post-deployment) as well as a DoD Abbreviated Concussion Symptom Inventory (ACSI). The ANAM4 TBI-MIL data set was provided by the Army Neurocognitive Assessment Branch, San Antonio, TX. The ACSI data set was provided by the Navy Bureau of Medicine and Surgery (BUMED) Wounded, Ill, and Injured Directorate (M9). After the datasets were cross-referenced 171 SMs met the inclusion criteria.

Exclusion criteria were if ANAM4-TBI-MIL scores were in the top 1% for speed while simultaneously in the bottom 1% for accuracy [6]. These criteria sought to exclude individuals who simply responded as quickly as possible to items without apparent attention to the accuracy of their response, which would lead to artificially faster response times with very poor accuracy, giving the false impression of superior performance [6]. After the exclusion criteria were applied to the datasets, 2 SMs were excluded, leaving the sample size at 169 SMs.

Available demographic for the total sample (Tables 1), prior to group assignment, indicated that 100% of the sample was male with a mean age of 22.5 years (SD ±3.4). The occupations of the Marines were primarily: rifleman (39%), infantry (20%), machine gunner (13%), mortarman (6%) and anti-tank missileman (4%). The remaining occupations were classified as “other” (17%).

**Table 1:** Sample characteristics (*n* = 169).

Characteristics		*n* (%)	Mean (SD)
*Age (y)*		-	22.5 (3.4)
*Deployments*	Afghanistan	163 (97)	1.1 (0.3)
	Iraq	27 (16)	1.5 (0.7)
*Concussion during*	Most recent deployment	59 (35)	1.2 (3.4)
	Previous deployment(s)	50 (30)	1.6 (0.8)
*Lifetime number of concussions*	0	85 (50)	1.8 (1.0)
	1	28 (17)	-
	2	17 (10)	-
	>2	5 (3)	-
	Total	152	10.8 (37.6)
*Blast exposure during*	Most recent deployment	146 (86)	7.0 (19.8)
	Previous deployment(s)	28 (17)	22.5 (74.4)
*Lifetime number of blast exposure*	0	17 (10)	-
	1	56 (33)	
	2	23 (14)	-
	>2	73 (43)	-
	Total		-
*Other event exposure*	Bullet	0 (0)	-
	Fragment	3 (2)	-
	Vehicle	12 (7)	-
	Sports	11 (7)	-
	Fall	14 (8)	-
	Fight	4 (2)	-
	Other	5 (3)	-
*Reporting*	Feeling “dazed, confused, saw stars”	73 (43)	-
	Loss of consciousness	27 (16)	-
	Posttraumatic amnesia	15 (9)	-

Pre-deployment (T1) data were from assessments that occurred at least six months preceding deployment. The initial Post-deployment (T2) data were from testing occurring two to eight weeks following return from deployment. The 2^nd^ post-deployment (T3) data were from testing that occurred from three to 18 months following the T2 post-deployment assessment. The T3 ANAM assessments come from those SMs who were preparing for another deployment to OIF or OEF and were done as part of DoD policy requiring pre-deployment neurocognitive testing. The average time (month ± SD) between testing was as follows: T1−T2 = 11±2.5, T1−T3 = 19±3.4 and T2−T3 = 7.6±2.5.

Group assignment (concussion and no concussion) was determined based on the results of a self-report TBI questionnaire administered as part of the ANAM4 TBI-MIL. Data from T2 was used in for group assignment. Individuals included in the concussion group reported an injury event accompanied by an alteration of consciousness. This included endorsement of at least one of the following symptoms immediately following the injury event: feeling dazed or confused, experiencing loss of consciousness, or experiencing loss of memory for the injury [6]. After group assessment, 76 and 93 SMs were assigned to the concussion and no concussion (control) groups, respectively.

### Instrument

The ANAM4 TBI-MIL is an automated, computerized neurocognitive assessment that includes a self-report TBI questionnaire, two subjective subtests (Sleepiness Scale and Mood Scale), and six performance subtests administered in order [6]. Detailed descriptions of these subtests can be found elsewhere [15]. For the purposes of this study, we analyzed the TBI questionnaire and the performance subtests: Simple Reaction Time (SRT), Procedural Reaction Time (PRO), Coded Substitution (CDS), Matching to Sample (M2S), Mathematical Processing (MTH), Code Substitution Delayed (CDD), and Simple Reaction Time Repeated (SRT2).

The ACSI is a self-report survey of blast exposure and eleven symptoms thought to be associated with TBI created by BUMED M9. The ACSI was designed as screening tool which would give medical providers mTBI specific symptomology to assist with the implementation of follow-on care.

### Dependent Variables

The ANAM4 TBI-MIL records accuracy, speed, and throughput performance on each subtest. Throughput (TP) is a single outcome measure produced from percent correct (accuracy) divided by mean reaction time (speed). Therefore, TP scores represent the correct number of responses per minute of available response time; thus, higher values indicate better performance [13]. TP is considered a measure of effectiveness or cognitive efficiency [16]. For the purposes of this study, only TP scores were used in the analyses.

### Statistical Analyses

Group differences in the combined ANAM4-TBI-MIL TBI questionnaire and ACSI symptomology were compared for the control and concussion groups for each testing session using three separate Wilcoxon rank sum tests with Sidak adjustments. Descriptive statistics were calculated on each ANAM4 TBI-MIL performance test. Data for each of the ANAM4 TBI-MIL performance tests were analyzed using a mixed model (2×3) analysis of variance (ANOVA) with repeated measures approach with group as the between-subjects variable and time as the within-subjects variable. A planned comparison for group [2 (group) x 2 (time) ANOVA with repeated measures] was performed between the baseline testing session and each of the post-test sessions (baseline vs. T2 and baseline vs. T3) for each performance test. If these results were significant, the analyses were followed by additional planned comparisons (paired *t*-tests) to isolate significance. Sidak adjustments were applied to the planned comparisons to maintain a family-wise alpha of .05. Analyses were performed with Matlab 2012b (Natick, MA).

## Results

There were significant differences in the total number of post-concussive clinical symptoms reported on the ANAM4-TBI-MIL TBI questionnaire and ACSI between the pre-deployment assessment and both post-deployment assessments across groups (Tables 2 and 3); however, there were no significant differences in number of post-concussive clinical symptoms reported between the two post-deployment assessments. The control group indicated that a total of 1 and 5 symptoms were worse from the baseline for T2 and T3, respectively. These differences may have been due to a deployment effect, (i.e., fatigue; Table 2). In contrast, the concussed group indicated that 20 and 21 symptoms were worse from the baseline for T2 and T3, respectively (Table 3).

**Table 2:** ANAM4 TBI-MIL Questionnaire and ACSI Symptomology for Non-Concussed Service Members (N = 93).

Symptom		T1		T2			T3					
		N	%	N	%	*W(T2 v T1)*	N	%		*W(T1 v T3)*		*W(T2 v T3)*
*Currently at Rest*												
	Headache	1	1%	3	3%	−1.74	8	9%		−3.24*		−2
	Nausea	1	1%	0	0%	0.00	0	0%		0.00		0.00
	Sensitivity to light	0	0%	0	0%	−0.99	4	4%		−2.01		−1.35
	Balance Problems	0	0%	0	0%	0.00	0	0%		−1.41		−1.41
	Ringing in Ears	1	1%	4	4%	−2.01	9	10%		−3.73*		−2.28
	Sleep Issues	0	0%	8	9%	−2.88	5	5%		−4.17*		−1.74
	Irritability	1	1%	3	3%	−1.00	10	11%		−2.79		−2.00
	Memory Loss	0	0%	2	2%	−1.41	4	4%		−2.48		−1.44
	Other	0	0%	2	2%	−0.57	2	2%		−1.41		0.00
*Currently after Exertion*												
	Headache	1	1%	2	2%	−0.57	10	0%		−2.38		−1.94
	Nausea	0	0%	1	1%	−0.99	4	4%		0.00		0.99
	Sensitivity to light	0	0%	1	1%	−0.99	2	2%		−2.01		−1.35
	Balance Problems	1	1%	2	2%	−0.57	13	14%		0.99		1.41
	Ringing in Ears	0	0%	3	3%	−1.00	16	17%		−2.59		−1.78
	Sleep Issues	0	0%	2	2%	−1.41	10	11%		−2.26		−1.15
	Irritability	0	0%	2	2%	−1.41	6	6%		−3.24*		−2.38
	Memory Loss	1	1%	4	4%	−1.35	2	2%		−1.35		0.00
	Other	0	0%	3	3%	0.08	8	9%		−1.41		0.45
*Symptom Total*		22	-	85	-	-	-		-		-	

Note: *p<.002. W =  Wilcoxon Rank-Sum Statistic. T1 =  pre-deployment. T2 =  post-deployment assessment. T3 = second post-deployment assessment.

**Table 3:** ANAM4 TBI-MIL Questionnaire and ACSI Symptomology for Concussed Service Members (N = 76).

Symptom		T1		T2			T3			
		N	%	N	%	*W(T2 v T1)*	N	%	*W(T1 v T3)*	*W(T2 v T3)*
*Right after Injury*										
	Headache	12	16%	62	82%	−9.88*	64	84%	−10.18*	−0.43
	Nausea	4	5%	23	30%	−5.39*	24	32%	−5.54*	0.86
	Sensitivity to light	4	5%	30	39%	−6.39*	34	45%	−6.95*	−0.65
	Balance Problems	7	9%	40	53%	−7.10*	48	63%	−8.22*	−1.31
	Ringing in Ears	5	7%	53	70%	−9.49*	56	74%	−9.90*	−0.54
	Sleep Issues	1	1%	36	47%	−6.98*	32	42%	−6.42	0.65
	Irritability	2	3%	30	39%	−6.66*	32	42%	−6.93*	−0.33
	Memory Loss	1	1%	35	46%	−7.08*	35	46%	−7.08*	0
	Other	2	3%	4	5%	−1.59	6	8%	−2.2	−0.65
*Currently at Rest*										
	Headache	3	4%	28	37%	−6.39*	22	29%	−5.54*	1.03
	Nausea	1	1%	1	1%	−1.09	3	4%	−1.92	−1
	Sensitivity to light	1	1%	14	18%	−4.31*	16	21%	−4.63*	−0.5
	Balance Problems	1	1%	7	9%	−2.98	7	9%	−2.98	0
	Ringing in Ears	2	3%	25	33%	−5.97*	28	37%	−6.39*	−0.51
	Sleep Issues	4	5%	26	34%	−6.11*	28	37%	−6.39*	−0.34
	Irritability	2	3%	28	37%	−6.11*	26	34%	−5.83*	0.34
	Memory Loss	2	3%	29	38%	−6.52*	27	36%	−6.25*	0.33
	Other	1	1%	1	1%	−1.09	3	4%	−1.09	0
*Currently after Exertion*										
	Headache	1	1%	20	26%	−4.93*	16	21%	−4.28	0.76
	Nausea	0	0%	4	5%	−2.23	3	4%	−1.92	0.38
	Sensitivity to light	0	0%	8	11%	−3.19*	10	13%	−3.59*	0.62
	Balance Problems	1	1%	5	7%	−1.91	10	13%	−3.15*	−1.35
	Ringing in Ears	0	0%	15	20%	−4.11*	18	24%	−4.61*	−0.59
	Sleep Issues	0	0%	11	14%	−3.78*	12	16%	−3.96*	−0.22
	Irritability	0	0%	20	26%	−5.25*	24	32%	−5.83*	−0.71
	Memory Loss	0	0%	18	24%	−4.61*	22	29%	−5.24*	−0.73
	Other	0	0%	1	1%	−1.09	1	1%	−1.09	0
*Symptom Total*		57	-	574	-	-	607	-	-	-

Note: *p<.002. W =  Wilcoxon Rank-Sum Statistic. T1 =  pre-deployment. T2 =  post-deployment assessment. T3 =  second post-deployment assessment

The mean pre-deployment and both initial and second post-deployment TP scores for service members with and without self-reported history of concussion are presented in Tables 4 and 5, respectively. ANOVA with repeated measures indicated that there were significant group by time interactions for SRT2 [*F*(2,334) = 5.95, *p* = 0.003], M2S [*F*(2,334) = 5.80, *p* = 0.003], CDS [*F*(2,334) = 7.05, *p*<0.000], and CDD [*F*(2,334) = 4.03, *p* = 0.019]. The follow-up ANOVA with repeated measures indicated that there was a significant group by time interaction between baseline testing and the post-deployment test for SRT2 [*F*(2,334) = 12.1, *p* = 0.000], M2S [*F*(2,334) = 6.83, *p* = 0.010], CDS [*F*(2,334) = 10.09, *p* = 0.002], and CDD [*F*(2,334) = 8.07, *p* = 0.005].

**Table 4:** Throughput Means (SE) for Non-Concussed Service Members.

PT	T1	T2	T3	Δ (T2-T1)	Hedge's *g*	Δ (T3-T1)	Hedge's *g*
*cdd*	48.3 (1.7)	53.3 (1.7)	50.8 (1.7)	5.0	−0.31	−2.5	−0.15
*cds*	54 (1.1)	55.1 (1.3)	56.5 (1.3)	1.1	−0.09	2.5	−0.21
*m2s*	32.8 (1.1)	32.0 (1.1)	32.8 (1.1)	−0.8	0.08	0.0	0.00
*mth*	20.1 (.7)	19.0 (.7)	20.6 (.7)	−1.1	0.17	0.5	−0.07
*pro*	96.7 (1.8)	98.8 (2.3)	95.8 (2.3)	2.1	−0.10	−0.9	0.05
*srt*	234.8 (3.9)	224.8 (4.8)	230.7 (4.8)	−10.0	0.24	−4.1	0.09
*srt2*	229.2 (4.5)	223.6 (5.1)	214.3 (5.5)	−5.6	0.12	−14.9	0.31

Note: No significant findings. PT =  performance test. T1 =  pre-deployment. T2 =  post-deployment assessment. T3 =  second post-deployment assessment. Δ (T2-T1) =  change from T1 to T2. Δ (T3-T1) =  change from T1 to T3. cdd =  coded substitution delayed. cds =  coded substitution. m2s =  matching to sample. pro =  procedural reaction time. srt =  simple reaction time. srt =  simple reaction time repeated.

**Table 5:** Throughput Means (SE) for Concussed Service Members.

PT	T1	T2	T3	Δ (T2-T1)	Hedge's *g*	Δ (T3-T1)	Hedge's *g*
*cdd*	51.3 (2.0)	45.5 (1.8)	49.1 (2.3)	−5.8	0.66	−2.2	0.41
*cds*	56.3 (1.4)	48.7 (1.3)	59.5 (1.7)	−7.6*	0.84	3.2	0.46
*m2s*	35.6 (1.4)	28.4 (1.1)	34.0 (1.5)	−7.2*	0.55	−1.7	0.05
*mth*	21.3 (1.0)	17.3 (.6)	20.9 (0.8)	−4.0	0.64	−0.4	0.13
*pro*	99.1 (2.0)	91.0 (2.5)	95.8 (2.6)	−8.1	0.40	−3.3	0.16
*srt*	237.5 (3.6)	206.8 (6.6)	217.9 (6.9)	−30.7	0.65	−19.6	−0.24
*srt2*	237.0 (4.2)	193.0 (7.3)	212.4 (7.5)	−44**	0.35	−24.6***	0.12

Note: SE =  standard error. PT =  performance test. T1 =  pre-deployment. T2 =  post-deployment assessment. T3 =  second post-deployment assessment. Δ (T2-T1) =  change from T1 to T2. Δ (T3-T1) =  change from T1 to T3. cdd =  coded substitution delayed. cds =  coded substitution. m2s =  matching to sample. pro =  procedural reaction time. srt =  simple reaction time. srt =  simple reaction time repeated. * *p* = 0.0001. **p = 0.000001. ***p = 0.005.

The planned comparison paired t-tests revealed that there were no significant differences between groups performance at baseline line testing and the second post-deployment test; however, there were significant differences revealed at the first post-deployment test for SRT2 [*t*(167) = 3.52, p = 0.001; *g* = 0.54]; M2S[*t*(167) = 3.44, p = 0.024; *g* = 0.35], CDS [*t*(167) = 3.44, p = 0.001; *g* = 0.53], and CDD [*t*(167) = 3.17, p = 0.002; *g* = 0.48].

The planned comparison paired t-tests revealed that there were no significant differences in control group performance (i.e., TP score) between the baseline and each of the post-deployment test sessions (i.e., control group performance was stable across all three testing sessions; Table 4). The paired t-tests revealed significant decrements in the concussion group performance from the baseline to the first post-deployment test SRT2 [*t*(150) = 5.21, p<0.000; *g* = 0.84], M2S [*t*(150) = 3.96, p<0.000; *g* = 0.63] and CDS [*t*(150) = 4.0, p<0.000; *g* = 0.65]. The significant decrement persisted to the second post-deployment assessment for SRT2 [*t*(150) = 2.86, p = 0.005; *g* = 0.46] only (Table 5).

Additionally, three out of the four performance tests were not found to be statistically significant. SRT, CDD, and MTH, all had a medium effect (Hedge's g>0.50). There, non-significant decrements in performance extended to the second post-deployment test session for all of the performance tests, albeit with small ES, except for CDS, which increased from the baseline session. It should be noted that these decrements were actually improvements from the first post-deployment test session and the only performance test that was statistically lower from the baseline was SRT2.

## Discussion

This is the first study to our knowledge that has examined longitudinal cognitive functioning in SMs with self-reported mTBI. Through the use of the ANAM4 TBI-MIL neurocognitive assessment during the post-concussive phase, we found declines in cognitive performance from the pre-deployment assessment (i.e., baseline) to the first post-deployment assessment (two to eight weeks after return from deployment) which, except for the second simple reaction time test, resolved by the second post-deployment assessment (∼seven months following first post-deployment assessment). This recovery pattern is consistent with the mTBI post-injury literature, as cognitive deficits in concussed individuals rarely last more than three months [13], [17]. However, the SMs reported an increase in post-concussive symptoms from baseline which did persist over time and did not resolve by the second post-deployment assessment.

These results suggest that cognitive declines during the chronic post-injury phase for some SMs with self-reported mTBI persist for periods as long as eight weeks post-deployment. This finding is contrary to previous studies on military samples which have reported that the ANAM4 TBI-MIL is only sensitive to cognitive declines during the acute post-injury phase (i.e., within 72 hours) and that there is no clinical utility outside of a ten-day post-injury window [18], [19]. However, other research has shown that cognitive deficits can persist for periods as long as one to three months, which is supported by our results [17], [19], [20]. Longitudinal studies that repeatedly assess cognitive performance in controlled intervals (i.e., on a monthly basis) will be necessary to better clarify recovery (or lack of recovery) patterns over time.

The only performance test that had persistent declines beyond the first post-deployment assessment was SRT2 in the concussion group. This result supports a growing body of literature that has identified impaired reaction time as the most sensitive marker of cognitive performance changes following an mTBI, and helpful in differentiating injured from non-injured SMs [10], [13], [19], [21], [22]. Impaired reaction time has been shown to have prognostic value (i.e., clinical utility) in predicting recovery especially when compared to a personal baseline rather than a group norm [23]. Reaction time is typically prolonged immediate post-injury and gradually returns back to baseline during recovery [22]. Decreases in reaction time have been suggested to be a result of injury to the anterior corona radiata and the uncinate fasciculus, as well as to the cingulum and the genu of the corpus callosum; however, normal interindividual variation in brain structure would require neuroimaging to isolate any neural correlate [24].

In the ANAM4 TBI-MIL, simple reaction time is currently scored based upon two administrations, one at the beginning (i.e., SRT) and one at the end (i.e., SRT2) of the battery.

Our results indicate that the second simple reaction time subtest (SRT2) is a more sensitive test longitudinally in differentiating injured from non-injured SMs. We propose that the poor performance on the SRT2 on both post-deployment tests separated by a period of several months may be an indicator of persistent cognitive/mental fatigue. Mental fatigue is a poorly understood symptom of mTBI where by fatigued individuals often report having difficulty focusing their attention and are, consequently, easily distracted [25−27]. Mental fatigue has been reported to be exacerbated by prolonged administration of neuropsychological testing [28]. We hypothesize that by the second post-deployment assessment, which is well into the chronic recovery phase, the SMs may have difficulties in cognitive functioning after finishing the entire test battery (i.e., 7 performance tests) due to the mental exertion required to complete it. Mental fatigue is may be exacerbated by post-injury sequelae such as pain, sleep difficulties, depression, and anxiety, all of which are often experienced by SMs post-deployment [25].

The SMs in this study experienced mTBI-related sequelae that persisted for an additional ∼5 months after most cognitive deficits resolved. Concussed SMs reported an increase in concussion-related symptoms from the baseline which persisted over time and had not resolved by the second post-deployment assessment. These results are also consistent with studies which have reported that symptoms are present six months or longer following deployment in SMs with mTBI [29]-[32] and/or emerge over time following deployment [33], [35], [36]. One study has reported that symptoms associated with self-reported mTBI were present one year post-deployment [34]. Persisting symptoms, however, are not specific to mTBI [33−36]. Thus, it is possible that the symptoms reported in the concussion group may actually be indicative of other conditions which could be co-morbid, such as post-traumatic stress disorder, depression, or pain, and that these factors may be the primary contributors to the observed declines in cognitive performance rather than persistent post-concussive syndrome.

A key limitation of the study was the high exclusion rate which is not a reflection of the clinical utility of the ANAM4-TBI-MIL test battery, but rather of DoD policy which mandates that all SMs undergo computerized neurocognitive testing prior to deployment to OEF and OIF. Current DoD policy does not mandate post-deployment testing. The assessments performed at T1 and T3 were performed prior to a deployment, as required by DoD policy. The assessments performed at T2 (i.e., post-deployment) were used to evaluate whether routine post-deployment ANAM testing should be implemented. The SMs who had a third ANAM assessment (T3) reflect a random selection from the initial sample - these were SMs who were transferred to new units that were chosen for deployment which necessitated an additional pre-deployment assessment (which is termed the 2^nd^ post-deployment assessment).

There were several additional limitations pertaining to this study. The SMs involved were members of units who had known high rates of concussion and blast exposure. Thus, the results of this study may not be generalizable to the general military or civilian populations. The data included self-reported symptom information; therefore, the results are subject to recall biases. The number of deployments was not controlled for, thus a deployment effect cannot be excluded as contributing factor. Another limitation of the current study is the ANAM4 TBI-MIL restriction to visually-based stimuli, which ignore other areas of cognition such as language skill and auditory processing. Clinicians who also assess performance in these additional areas could provide a more comprehensive clinical evaluation; however, a lengthier test battery is not practical for the combat zone. The use of neurocognitive testing in the absence of clinical evaluation should be questioned as psychological functioning (i.e., depression) which can be a major confounder of cognitive functioning and, thus, test results [35]. A normal limitation of neurocognitive performance is affected by age, ethnic background, education, and developmental disorders; however, comparing post-injury scores to a baseline results in greater diagnostic accuracy than comparison to a group normative value [11]. An additional limitation was that there were four databases which were cross-referenced to ensure that SMs had data for each ANAM4-TBI-MIL assessment and for the ACSI. Not all service members had data for three assessments which led to a high exclusion rate of 1152 SMs. As cognitive functioning returned to baseline levels while symptoms persisted, these results highlight the need for a detailed clinical examination in the setting of a history of concussion where there are persistent clinical symptoms, rather than relying solely upon computerized cognitive testing as a method for identifying SMs requiring follow-up post-injury. Our results do indicate that there is a measurable deployment effect on neurocognitive performance, which appears to resolve without lasting clinical sequelae in those not injured. In those who sustained a concussion, the most striking late finding is the presence of persistent clinical symptoms and an isolated persistent impairment in simple reaction time but no other detectable impairment of cognitive test measures.
